# Assessing the appropriateness of paediatric antibiotic overuse in Australian children: a population-based sample survey

**DOI:** 10.1186/s12887-020-02052-6

**Published:** 2020-04-24

**Authors:** Gaston Arnolda, Peter Hibbert, Hsuen P. Ting, Charli Molloy, Louise Wiles, Meagan Warwick, Tom Snelling, Nusrat Homaira, Adam Jaffe, Jeffrey Braithwaite, on behalf of the CareTrack Kids investigative team

**Affiliations:** 1 Australian Institute of Health Innovation, Macquarie University, Level 6, 75 Talavera Road, North Ryde, New South Wales 2109 Australia; 2 Australian Centre for Precision Health, University of South Australia Cancer Research Institute, University of South Australia, Adelaide, South Australia Australia; 3 Perth Children’s Hospital, Nedlands, Western Australia Australia; 4 Faculty of Medicine, University of New South Wales, Sydney, New South Wales Australia; 5 Respiratory Department, Sydney Children’s Hospital, Sydney, New South Wales Australia

**Keywords:** Antibiotic, Overuse, Guideline adherence, Appropriate

## Abstract

**Background:**

Infections caused by antibiotic resistant pathogens are increasing, with antibiotic overuse a key contributing factor.

**Objective:**

The CareTrack Kids (CTK) team assessed the care of children in Australia aged 0–15 years in 2012 and 2013 to determine the proportion of care in line with clinical practice guidelines (CPGs) for 17 common conditions. This study analyses indicators relating to paediatric antibiotic overuse to identify those which should be prioritised by antimicrobial stewardship and clinical improvement programs.

**Method:**

A systematic search was undertaken for national and international CPGs relevant to 17 target conditions for Australian paediatric care in 2012–2013. Recommendations were screened and ratified by reviewers. The sampling frame comprised three states containing 60% of the Australian paediatric population (South Australia, New South Wales and Queensland). Multi-stage cluster sampling was used to select general practices, specialist paediatric practices, emergency departments and hospital inpatient services, and medical records within these. Medical records were reviewed by experienced paediatric nurses, trained to assess eligibility for indicator assessment and compliance with indicators. Adherence rates were estimated.

**Results:**

Ten antibiotic overuse indicators were identified; three for tonsillitis and one each for seven other conditions. A total of 2621 children were assessed. Estimated adherence for indicators ranged from 13.8 to 99.5% while the overall estimate of compliance was 61.9% (95% CI: 47.8–74.7). Conditions with high levels of appropriate avoidance of antibiotics were gastroenteritis and atopic eczema without signs of infection, bronchiolitis and croup. Indicators with less than 50% adherence were asthma exacerbation in children aged > 2 years (47.1%; 95% CI: 33.4–61.1), sore throat with no other signs of tonsillitis (40.9%; 95% CI: 16.9, 68.6), acute otitis media in children aged > 12 months who were mildly unwell (13.8%; 95% CI: 5.1, 28.0), and sore throat and associated cough in children aged < 4 years (14.3%; 95% CI: 9.9, 19.7).

**Conclusion:**

The results of this study identify four candidate indicators (two for tonsillitis, one for otitis media and one for asthma) for monitoring by antibiotic stewardship and clinical improvement programs in ambulatory and hospital paediatric care, and intervention if needed.

## Background

There have been substantial concerns relating to antibiotic overuse in recent decades. Antibiotic overuse can accelerate the rate of development of antibiotic resistance [1], and contribute to wasteful misuse of limited health resources [2–4]. The World Health Organization recognises emergence of antimicrobial resistance as a threat to global and national security and has expressed concern about its impact on the effectiveness of health programs [5]. Detailed estimates of national or international costs associated with antibiotic resistance are not published. However, the results of a single hospital study [6] have been extrapolated to estimate a total medical cost attributable to antimicrobial-resistant infection of $20 billion in the United States of America, with an additional $35 billion in broader societal costs [7]; this result is promulgated in a report by the US Centers for Disease Control [8] and was subsequently criticised as seriously underestimating the scale of the problem [9].

The extent of overuse drives the concern about emerging resistance. In Australia, General Practitioners (GPs) are estimated to be prescribing between four and nine times as many antibiotics as would be expected for acute respiratory tract infections (RTIs) if they were following published guidelines [10]. For paediatric patients, GP prescribing rates were also found to be above that recommended by guidelines for upper RTIs, bronchitis and tonsillitis [11]. In the US, 40% of children undergoing procedures not requiring perioperative antibiotic prophylaxis were inappropriately given an antibiotic [12]. In the Netherlands and Israel, up to a third of all children with a lower respiratory tract infection due to respiratory syncytial virus were unnecessarily treated with antibiotics [13].

A variety of drivers of antibiotic overuse have been proposed, including: that parents have expectations for prescribed antibiotics for mild infections [14]; that health professionals’ perceptions of these expectations influence their prescribing practices [15]; and that the community lacks knowledge about the emergence of antibiotic resistant strains, and the risks they pose [16]. In addition, there is evidence that some physicians and pharmacists believe antibiotics can reduce complications of mild illnesses such as common cold [17], despite evidence to the contrary [18]. Given the variety of interacting factors, studies are needed that examine how they shape actual prescribing behaviour in real world contexts, to inform interventions [19]. For example, a systematic review of qualitative studies directly observing care has noted that primary care clinicians may be misinterpreting parent requests for information as requests for antibiotic prescription, suggesting novel possible interventions at clinician level, to address this [20].

To identify intervention targets for unnecessary antibiotic use it is important to first identify conditions where antibiotics are used regularly but without known benefit. The CareTrack Kids (CTK) team assessed care of Australian children aged 0–15 years in 2012 and 2013 to determine the proportion who received care in line with clinical practice guidelines (CPGs) for 17 common conditions [21]. Across the 17 conditions, guideline-adherent care was provided for 59.8% (95% CI: 57.5–62.0) of indicators. Here, we present and discuss the CareTrack Kids results for indicators specifically relating to antibiotic overuse, found in eight of the 17 conditions, to help identify potential candidates for intervention.

## Methods

The CTK methods have been described in detail, elsewhere [21, 22]. We describe some aspects specifically relevant to an analysis of indicators relating to antibiotic overuse.

### Development of indicators

We modified and applied the RAND-UCLA method to develop indicators [23]. A clinical indicator was defined as a measurable component of a standard or guideline, with explicit criteria for inclusion, exclusion, time frame and practice setting [24].

A systematic search for Australian and international CPGs relevant for the years 2012–2013 yielded 99 CPGs for clinical conditions under consideration, from which 1266 recommendations were extracted. We screened recommendations for eligibility and excluded based on four criteria: (1) their strength of wording (“may” and “could” statements were excluded); (2) a low likelihood of the information being documented; (3) guiding statements provided without recommended actions; and (4) aspects of care deemed out of scope of the CTK study such as “structure-level” recommendations; 322 recommendations were excluded, with the remaining 944 recommendations used to draft initial indicators. The recommendations were converted into a structured and standardised indicator format [22]. They were then assigned the type of quality care addressed (underuse; overuse).

Proposed indicators were ratified by experts over a two-stage multi-round modified Delphi process, which comprised an email-based three-round internal review and a collaborative, online, wiki-based two-round external review, custom-designed for the study [22]. In total, 146 experts (including 104 pediatricians and 22 general practitioners (GPs)) were recruited for the internal (*n* = 55) and external review (*n* = 91) [25]. A clinical expert was appointed to lead the reviews for each condition. Reviewers completed a Conflict of Interest declaration [22], using an established protocol [26], and worked independently to minimise group-think [27].

In the internal review, experts scored each indicator against three criteria; acceptability, feasibility and impact [22]; recommended indicators for inclusion or exclusion; and provided any additional comments. For the external review, experts registered to the online wiki and self-nominated for CTK conditions based on their clinical experience [25]. External reviewers applied the same scoring criteria as internal reviewers and, in addition, used a nine-point Likert scale to score each indicator as representative of appropriate care delivered to children during 2012 and 2013 [22, 23]. The clinical expert for each condition commented on reviewers’ responses, and made final recommendations regarding the inclusion of the indicators. A total of 479 final indicators were ratified by experts and grouped, creating 17 condition-specific surveys; of these, ten were antibiotic overuse indicators, drawn from eight conditions. All indicator questions relating to antibiotic overuse are shown in Table 1.

**Table 1:** Characteristics of antibiotic overuse indicators and number of sites sampled, 2012–2013

				No. of Sites				
Condition	IndicatorID	Indicator Description	Age Inclusion Criteria	GP	Paed-iatrician	ED	Inpatient	Strength of Recommendation^**a**^
Acute Gastroenteritis	AGE22	Children with gastroenteritis and no signs of infection were not prescribed antibiotics.	0–15 years	74	NA	34	26	Consensus-based recommendation
Asthma	ASTH16	Children aged > 2 years who presented with an acute exacerbation of asthma and who received antibiotics had another condition requiring antibiotic therapy.	2–15 years	40	1	19	10	Consensus-based recommendation
Bronchiolitis	BRON25	Infants (aged < 12 months) with mild to moderate bronchiolitis caused by a viral infection were not prescribed antibiotics.	29 days - 11 months	54	NA	33	27	Grade B
Croup	CROU16	Children diagnosed with croup were not treated with antibiotics.	29 days - 15 years	71	NA	34	23	Consensus-based recommendation
Eczema	ECZE07	Children with atopic eczema and no signs of infection were not prescribed antibiotics.	0–15 years	72	18	23	10	Grade B
Fever	FEVE29	Children aged ≥3 years with a fever (over 38 °C), no clinical focus and who were well were not prescribed antibiotics.	3–15 years	32	#	27	7	Consensus-based recommendation
Otitis Media	OTIT05	Children with AOM aged ≥12 months who were mildly unwell were not prescribed antibiotics.	1–15 years	75	3	30	3	Grade B
Tonsillitis	TONS02	Children with a sore throat and with no other symptoms or signs of tonsillitis were not prescribed antibiotics.	29 days - 15 years	43	#	23	5	Grade A
	TONS04	Children aged < 4 years with a sore throat and associated cough who did not require hospitalisation were not prescribed antibiotics.	29 days - 3 years	51	#	25	NA	Consensus-based recommendation
	TONS07	Children who had a tonsillectomy and adenoidectomy were not administered perioperative antibiotics.	29 days - 15 years	NA	NA	NA	5	Consensus-based recommendation

Legend: *ID* identifier, *GP* general practitioner, *ED* emergency department, *AOM* acute otitis media

^a^Strength of recommendation as reported in individual clinical practice guidelines (CPGs). CPGs used a variety of classification schemes for allocating Strength of Recommendation in Grades (with Grade A indicating the strongest recommendation in all classification schemes). If strength of recommendation, or Level of Evidence, were not specified in the CPG, the term “Consensus-based recommendation” was assigned

^#^ Specialist Paediatrician’s practices were sampled for visits for care of fever and tonsillitis, but only one and three records were found respectively, so this healthcare setting was removed prior to analysis

### Sample size, sampling process and data collection

CTK identified 6689 medical records for 17 conditions. If any of the 6689 medical records we sampled contained a visit for any of the 17 conditions, a separate assessment of appropriateness was made for each visit. Detail on the general sampling methods are provided elsewhere [21]; additional details relevant to antibiotic overuse can be found in the Additional file 1. Briefly, we sampled four health care settings: hospital inpatients and emergency department (ED) presentations, and consultations with GPs and paediatricians in randomly-selected health department administrative units (Health Districts) in Queensland, New South Wales and South Australia, for children aged ≤15 years receiving care in 2012 and 2013. Data were collected by nine experienced paediatric nurses (“surveyors”), trained to assess eligibility for indicator assessment and compliance with CPGs.

### Analysis

At indicator level, estimates of compliance were measured as the percentage of eligible indicators (i.e., indicators answered either ‘Yes’ or ‘No’) which were scored as ‘Yes’. Weights were constructed to reflect the estimated incidence of presentation of each condition in each sampling unit separately for each setting, as briefly outlined in the Additional file 1, and fully detailed in the Additional file 1 to the report of top-level study results [21]. The overall overuse estimates were calculated as the weighted average of the relevant indicators, as a group, with weights taking into account the relative incidence of presentation for each condition (see Additional file 1); an overall estimate was also calculated for three tonsillitis indicators. The weighted data were analysed in SAS v9.4, using the SURVEYFREQ procedure. Variance was estimated by Taylor series linearization. State and setting were specified as strata, and the primary sampling unit (Health District) was specified as the clustering unit. Exact 95% CIs were generated using the modified Clopper–Pearson method. Indicator results were suppressed if there were < 25 eligible visits, but these results contributed to overall results by overuse, and by condition.

### Ethical considerations

We received primary ethics approval from relevant bodies including the Royal Australian College of General Practitioners (NREEC 14–008) and state hospital networks (HREC/14/SCHN/113; HREC/14/QRCH/91; HREC/14/WCHN/68), and site-specific approvals from 34 hospitals. Australian human research ethics committees can waive requirements for patient consent for external access to medical records if the study entails minimal risk to HCPs and patients [22]; all relevant bodies provided this approval. Participants were protected from litigation by gaining statutory immunity for CTK as a quality assurance activity, from the Federal Minister for Health under Part VC of the Health Insurance Act 1973 (Commonwealth of Australia).

## Results

### Characteristics of surveyed medical records and HCPs

Details of the 2621 children with one or more eligible assessments of CPG compliance for antibiotic overuse are provided in Table 2. About half the children were aged under 3 years, with slightly more males than females.

**Table 2:** Characteristics of the eligible children, 2012–2013

Characteristic	Children with antibiotic overuse indicators				
	GP (*n* = 1510)	Paediatrician (*n* = 69)	ED (*n* = 987)	Inpatient (*n* = 271)	Total^**a**^ (*n* = 2621)
Age^b^ - no. (%					
< 1 years	260 (17.2)	33 (47.8)	261 (26.4)	111 (41.0)	574 (21.9)
1–2 years	426 (28.2)	19 (27.5)	313 (31.7)	63 (23.2)	772 (29.5)
3–4 years	305 (20.2)	9 (13.0)	172 (17.4)	37 (13.7)	492 (18.8)
5–11 years	440 (29.1)	5 (7.2)	204 (20.7)	48 (17.7)	660 (25.2)
12–15 years	79 (5.2)	3 (4.3)	37 (3.7)	12 (4.4)	123 (4.7)
Male – no. (%)	787 (52.1)	43 (62.3)	583 (59.1)	157 (57.9)	1444 (55.1)

Legend: *GP* general practitioner; *ED* emergency department

^a^Total is less than the sum of the individual health care settings, as 216 children had both ED and inpatient visits

^b^The child’s age was calculated as the age at visit where there was only one, or the midpoint of the child’s age at her first and last visit with an antibiotic overuse indicator assessment, where there was more than one

Of 12,562 possible antibiotic overuse indicator assessments, 3237 (25.8%) were automatically filtered out by age or setting restrictions, and surveyors designated a further 5344 (42.5%) as not applicable or otherwise ineligible. Surveyors conducted 3981 indicator assessments during 3935 visits. Eligible antibiotic overuse assessments were conducted in 81 GP and 18 paediatric practices, 34 hospital EDs and 29 hospital inpatient services.

### Adherence

The estimated adherence for each of the 10 overuse indicators is shown in Table 3; appropriateness is not reported for one indicator (TONS07), as it was measured in < 25 visits. For the nine indicators with reported data, compliance ranged from 13.8% for indicator OTIT05 (“*Children with acute otitis media aged > 12 months who were mildly unwell were not prescribed antibiotics.*”) to 99.5% for ECZE07 (“*Children with atopic eczema and no signs of infection were not prescribed antibiotics*.”). Estimated adherence across the ten indicators was 61.9% (95% CI: 47.8–74.7).

**Table 3:** Appropriateness of care, for antibiotic overuse indicators, 2012–2013

Condition	Indicator ID	Indicator description	No. of Children	No. of Visits	Proportion Adherent % (95% CI)
Acute Gastroenteritis	AGE22	Children with gastroenteritis and no signs of infection were not prescribed antibiotics.	604	757	97.8 (95.6, 99.1)
Asthma	ASTH16	Children aged > 2 years who presented with an acute exacerbation of asthma did not receive antibiotics unless they had another condition requiring antibiotic therapy^a^	119	150	47.1 (33.4, 61.1)
Bronchiolitis	BRON25	Infants (aged < 12 months) with mild to moderate bronchiolitis caused by a viral infection were not prescribed antibiotics.	334	484	86.1 (74.8, 93.7)
Croup	CROU16	Children diagnosed with croup were not treated with antibiotics.	724	973	84.5 (59.2, 97.1)
Eczema	ECZE07	Children with atopic eczema and no signs of infection were not prescribed antibiotics.	499	622	99.5 (98.5, 99.9)
Fever	FEVE29	Children aged > 3 years with a fever (over 38°), no clinical focus and who were well were not prescribed antibiotics.	111	126	78.8 (51.1, 95.0)
Otitis Media	OTIT05	Children with AOM aged > 12 months who were mildly unwell were not prescribed antibiotics.	417	487	13.8 (5.1, 28.0)
Tonsillitis	TONS02	Children with a sore throat and with no other symptoms or signs of tonsillitis were not prescribed antibiotics.	144	159	40.9 (16.9, 68.6)
	TONS04	Children aged < 4 years with a sore throat and associated cough who did not require hospitalisation were not prescribed antibiotics.	179	216	14.3 (9.9, 19.7)
	TONS07	Children who had a tonsillectomy and adenoidectomy were not administered perioperative antibiotics.	7	7	Insufficient data
Overall overuse indicators			2621	3935	61.9 (47.8, 74.7)

Legend: *AOM* acute otitis media

^a^The wording of the original indicator has been amended for clarity and consistency with other indicators – the original wording can be found in Table 1

The overall estimate for compliance with antibiotic overuse indicators masks substantial heterogeneity. The two overuse indicators with near perfect compliance were for children with gastroenteritis or eczema without signs of infection (AGE22 [97.8%; 95% CI: 95.6, 99.1] and ECZE07 [99.5%; 95% CI: 98.5, 99.9], respectively). Overuse indicators for mild to moderate bronchiolitis (BRON25; 86.1%; 95% CI: 74.8, 93.7), croup (CROU16; 84.5; 95% CI: 59.2, 97.1) and fever with no clinical focus aged > 3 years (FEVE29; 78.8; 95% CI: 51.1, 95.0) all had moderately high levels of compliance.

Overuse indicators for asthma exacerbation (ASTH16; 47.1%; 95% CI: 33.4–61.1) and sore throat (TONS02; 40.9%; 95% CI: 16.9, 68.6) had poor compliance, under 50%. The vast majority of children with acute otitis media who were mildly unwell (OTIT05; 13.8%; 95% CI: 5.1, 28.0), and children with sore throat and associated cough (TONS04; 14.3%; 95% CI: 9.9, 19.7) received antibiotics contrary to recommendations. As there were three indicators of antibiotic overuse for tonsillitis, we calculated an overall estimate across the three indicators of 24.3% (95% CI: 16.1–34.2).

## Discussion

Our overall estimate of compliance with antibiotic overuse indicators was 61.9% (95% CI: 47.8–74.7) across 10 indicators. This estimated rate of adherence to guidelines advocating against antibiotic overuse was markedly lower than that found for *all* overuse indicators in the broader CTK study (87.2% compliance; 95% CI: 80.7–92.1), which included overuse of other tests and treatments [21]. There was substantial variation in adherence rates across indicators.

First, we consider indicators with higher compliance. In relation to antibiotic use for eczema without signs of infection (99% adherence) or fever in well infants aged > 3 years (79%) we are not aware of other studies reporting adherence rates to which we can compare. For gastroenteritis, a study of Welsh GPs found only 2.4% rate of antibiotic use for children [28], reflecting the 98% compliance in our study, confirming that this is not a priority for intervention. A study of inpatient admissions for bronchiolitis at a single US hospital found rates of antibiotic use of 27% before introduction of a guideline reducing to 9% after introduction [29]. The CTK indicator was restricted to children with mild to moderate bronchiolitis and included ambulatory settings and had 14% antibiotic use (i.e., 86% adherence). While the US study results are encouraging in demonstrating the potential for improvement in a single hospital, reducing antibiotic use across a variety of sites and settings is a more complex endeavour.

For indicators with lower adherence, published research provides some insights without directly assessing the indicators measured in our study. For asthma, an assessment of the management of acute exacerbations in children and adolescents found that antibiotic use was only half as frequent in specialist paediatric EDs as it was in general EDs [30], suggesting greater comfort with non-use of antibiotics in the more specialist setting. There was a 53% rate of antibiotic use in the CTK study, which included ambulatory settings, possibly indicating the need for greater support to be provided to less specialised settings, including primary care.

The CTK study found an 86% rate of antibiotic prescription for children with acute otitits media (AOM) who were mildly unwell (i.e., 14% compliance), across ambulatory and non-ambulatory patients. This result may reflect that there is no consensus surrounding the use of antibiotics vs expectant management in the management of mild AOM. Importantly, the CTK study did not distinguish between antibiotics prescribed for immediate use and those prescribed as a ‘back-up’ to allow treatment to commence promptly, if required; both would have been classified as non-compliant. Australia’s move to nationally funded universal coverage with the pneumococcal vaccine for infants in 2005 [31], following targeted vaccination introduced in 2001, does not appear to have markedly reduced the propensity to use antibiotics for AOM. GP survey data show that the rate of prescription of antibiotics for OM was 84% in 2003–2007 and 80% in 2011–2015 [32]. Similarly, US data suggests little change following guideline publication [33]. Concern about antibiotic prescribing practice for AOM continues in the US where the local ‘Choosing Wisely’ campaign recommends observation over antibiotics for AOM, where feasible [34].

Our study found a 59% rate of antibiotic prescription for sore throat on its own, and an 86% antibiotic prescription rate for sore throat and cough in children aged < 4 years. The results for sore throat and cough broadly agree with Australian GP survey data for ‘tonsillitis’ in children aged < 5 years (89% prescribed antibiotics); data for diagnosis of ‘throat symptoms/complaint’ was not examined in this study [11].

Given the many decades of concern about antibiotic overuse, our findings for asthma, sore throat and otitis media were disappointing. A number of potential reasons for antibiotic overprescribing have been suggested, including clinical time constraints, diagnostic uncertainty, risk aversion associated with fear of litigation, patient health beliefs and literacy and the knowledge, skill and attitude of clinicians [35–38]. It has also been suggested that the broader context of antibiotic prescribing needs to be taken into account to understand non-compliance, including clinician perceptions of what is required to sustain a longer-term clinician-patient relationship and social norms [19].

Soundly executed interventions can improve compliance with antibiotic guidelines. A review of clinician-targeted interventions to reduce antibiotic prescribing for acute respiratory infections in primary care found benefits – particularly, those interventions targeting point of care testing for C-reactive protein (22% reduction), shared decision-making (56% reduction) and procalcitonin-guided management (90% reduction). The quality of evidence for interventions focused on clinician educational materials and decision support was too poor to confidently assess [39]. Training Canadian family physicians in shared decision-making strategies for 2 hours online, followed by a 2-hour interactive seminar, reduced the risk of antibiotic overuse in children by 60% [40]. A recent Australian review identified a number of promising interventions, including audit and feedback, personalised letters to high prescribers, delayed prescribing, shared decision-making, and near-patient diagnostic testing for CRP and procalcitonin [41]. A consensus for the need of multi-faceted interventions targeting providers, patients, and the public and incorporating behavioural or psychosocial interventions, outpatient stewardship, and judicious clinicians is growing in the literature [42–44].

### Strengths and weaknesses

The strengths and weaknesses of the CTK study are described in additional detail elsewhere [21], and are summarised briefly here. For logistical reasons, we restricted our coverage to larger hospitals providing ~ 40% of all inpatient and ED care in the chosen geographies. While hospitals had excellent participation rates, the participation rates of GPs and specialist paediatricians is estimated at around 25% (see Additional file 1); it is plausible that self-selection has led to our study over-estimating compliance. Within health care sites, random record selection was externally controlled in both hospital and GP settings, but could not be standardised in paediatrician consulting rooms, with unknown impacts on our estimates of compliance. Finally, our study assessed documented practice, and it is plausible that this differs from actual practice; in primary care, it has been estimated that this can lead to underestimation of overall compliance by around 10 percentage points [45]. On the other hand, some antibiotic prescriptions may be provided without being filled.

There are also strengths and weaknesses which are specific to our assessment of antibiotic overuse in the present study. The study was not designed to assess compliance with CPGs which address antibiotic overuse overall; rather, we assessed compliance with CPG indicators associated with 17 clinical conditions, ten of which were about antibiotic overuse. The overall estimates of compliance with overuse indicators can only therefore be generalised to these conditions. As a strength, however, it should be noted that most studies examining antibiotic use are restricted to reporting prescribing rates, without an assessment of compliance with a specific CPG; our study, by contrast, trained experienced paediatric nurses to assess compliance with specific CPGs.

### Implications

Our results help to identify conditions with high levels of antibiotic overuse in paediatric settings. The methods we used can be adapted to measure non-adherent antibiotic prescribing for a broader range of clinical conditions, to prioritise targets for intervention and increase guideline compliance. Such studies should consider distinguishing between prescriptions provided for immediate filling and those provided in case of deterioration. This information can be used by existing antimicrobial stewardship programs and clinical improvement programs in primary care, to prioritise targets for intervention.

## Conclusion

There is a need to achieve substantial and sustained reductions in over prescription of antibiotics. Our study identified four presentations with > 50% antibiotic prescription, contrary to guidelines: AOM in mildly unwell children aged > 12 months; children with sore throat and cough in children aged < 4 years; children with sore throat and no other signs of tonsillitis; and children aged > 2 years presenting with an acute exacerbation of asthma.

## Supplementary information

**Additional file 1.** Additional details relating to study methods.

## Data Availability

Patient data in this study are not publicly available as they were collected from medical records examined by the research team without seeking individual consent. Four ethics committees approved this data extraction without consent and would need to approve the release of data collected by the project, to ensure protection of both healthcare providers and individual patients. Most of the data used for calculation of weights is owned by third parties, and its release will be subject to third party approvals from: three state health departments (populations by health district, total ED presentations and inpatient admission numbers by hospital, percentage of ED admissions by condition), the Australian Government Department of Human Services (total number of consultations with children by General Practitioners and community paediatricians), the Australian Paediatric Research Network (percentage of consultations for each condition by community paediatricians) and the Bettering the Evaluation and Care of Health Program (percentage of consultations by condition for General Practice). Data will be made available by the authors upon reasonable request, and with the approval of all bodies from whom permissions are required.

## Abbreviations

AGE22: Identifier for acute gastroenteritis indicator
AOM: Acute otitis media
ASTH16: Identifier for asthma indicator
BRON25: Identifier for bronchiolitis indicator
CPG: Clinical practice guideline
CROU16: Identifier for croup indicator
CTK: CareTrack Kids study
ECZE07: Identifier for eczema indicator
ED: Emergency department
FEVE29: Identifier for fever indicator
GP: General practitioner
OTIT05: Identifier for otitis media indicator
RTI: Respiratory tract infection
SURVEYFREQ: Name of SAS procedure used for analysing sample survey data
TONS02, TONS04 and TONS07: Identifiers for tonsillitis indicators
