# Supplementary material for: Assessing the appropriateness of paediatric antibiotic overuse in Australian children: a population-based sample survey
Source: BMC Pediatr. 2020 Apr 24;20:185. doi: 10.1186/s12887-020-02052-6 (PMC7181474; doi:10.1186/s12887-020-02052-6)
Supplement: Supplementary file 1 — Additional file 1. Additional details relating to study methods. [file 12887_2020_2052_MOESM1_ESM.docx]

**Appendix: Additional details relating to study methods**

The report of top-level CareTrack Kids CTK results[1] and its associated online appendix detail the methods of the larger study, which generated the data reported in this paper. Selected methods specifically relevant to antibiotic overuse are described below.

**Sample size**

A visit, during which multiple indicators may be assessed, was defined as an occasion of admitted inpatient care, an Emergency Department (ED) presentation or a consultation at a General Practice (GP) or a paediatrician in a private practice role. Without adjustment for the design effect, a minimum of 400 indicators for each of 16 conditions was required to obtain national estimates with 95% Confidence Interval (CI) and precision of +/- 5% at condition level. Anxiety and Depression as a single condition for sampling purposes.

No sample size was specified for antibiotic overuse. Rather, this study represents a post hoc analysis of data focusing on indicators relating to antibiotic overuse. There were 10 such indicators spread across eight conditions (‘target conditions’), so the total number of records targeted for conditions containing an antibiotic overuse indicator was 3,200. Not all of these records contained a visit where an antibiotic indicator needed to be assessed. Conversely, if a record sampled for a condition not containing an antibiotic overuse indicator happened to contain a visit for a condition with an antibiotic overuse indicator, all visits were reviewed and any eligible antibiotic overuse indicator was assessed. In brief, the sample size was unknown.

**Sampling** **Process**

A multistage stratified random sampling process was implemented. For logistical efficiency, sampling was targeted at three states, Queensland (QLD), New South Wales (NSW) and South Australia (SA), which together comprise 60.0% of the estimated Australian population aged 15 years or younger in the 2012 and 2013 calendar years. All six paediatric tertiary hospitals (two in QLD, three in NSW, and one in SA) were targeted as they have state-wide coverage. State Departments of Health organize care within administrative units (health districts): Hospital Health Services in QLD, Local Health Districts in NSW, and Local Health Networks in SA. For QLD, we targeted five health districts (two metropolitan, three regional), in NSW four health districts (two metropolitan, two regional), and in SA three health districts (two metropolitan, one regional). Despite best efforts, paediatricians were not recruited in targeted health districts in SA, so they were recruited in a metropolitan health district that was not randomly selected.

**Recruitment of health care providers**

Within the selected health districts, we approached all public hospitals, or private hospitals providing public services under contract, that had patient volumes of ≥2,000 ED presentations and ≥500 paediatric separations per year; we also advertised the study to general practices and paediatricians, and approached all the providers we could identify through internet searches, and via personal contacts. Within the selected sites, we sampled medical records for each condition targeted at that setting.

As noted in the main text, 34 of 37 (92%) eligible hospitals that were approached agreed to participate. Recruitment of GPs and paediatricians was decentralized. Administrative details for refusal rates, from cold-calling or direct contact by clinicians who facilitated recruitment of their peers, were maintained on project laptops. At the end of recruitment all computers were decommissioned and cleaned, with the files archived on a USB. Unfortunately, the USBs created during laptop decommissioning were misplaced and have not been able to be located. This did not affect the quality indicator adherence data, as the database was remotely located and updated regularly via the internet. We have therefore sought to estimate the recruitment rates based on recruitment spreadsheets emailed to the administrative staff.

For GPs, we were only able to locate emailed spreadsheets with late stage records for one state, South Australia. Based on this spreadsheet, we approached 114 GPs and recruited 27 of them, giving a recruitment rate of 23.7%; an additional GP, not listed on the available spreadsheet, was recruited subsequently and was not added to either the numerator or the denominator, for this estimate. The spreadsheet did not have clear information on eligibility, so it is likely that an unknown number of the 114 approached were ineligible because: 1) they were not open during the whole 2012-2013 survey period; 2) they saw no or few children; or 3) they were not confident in their ability to generate full listings of children with the target conditions, or they did not use one of the four practice software systems our surveyors were trained to search. Our estimate of 23.7% is therefore likely to be an underestimate of the actual recruitment rate.

For paediatricians, we were fortunate to be able to locate emailed records with late stage records for all three states. Based on these spreadsheets, we successfully approached 80 eligible paediatricians and recruited 20 of them, giving a recruitment rate of 25.0%.

Self-selection of GPs and paediatricians, and the estimated 24-25% recruitment rate, could lead to bias in the estimated guideline adherence, arising from self-selection. It is plausible that self-selected practices were more confident of their guideline adherence, potentially leading to overestimation of the quality of care in the CareTrack Kids study.

**Allocation of surveys to sampling units**

The number of records per condition targeted at each site was determined by a nominal allocation of the 400 records targeted, informed by data available at the time, supplemented by expert opinion, with planned over-sampling of settings where fewer occasions of care were expected.[1, 2] For hospitals, a fixed number was targeted at each site; for paediatricians, a fixed number was targeted initially, but this was abandoned as it was not possible to systematically identify patients by condition; for GPs, different combinations of conditions were targeted at each site, to simplify the logistics of sampling.

**Data collection**

Nine experienced paediatric nurses were employed across the three states, with all nine assessing occasions of care for antibiotic overuse. The surveyors undertook a one-week training program, prior to data collection. A surveyor manual was developed which included instructions, condition-specific definitions, inclusion and exclusion criteria, and guidance for assessing eligibility of each encounter for relevant indicators.

A web-based tool, originally developed for the CareTrack Adults study,[3, 4] was designed to enter data during medical record review. Algorithms to filter indicators by HCP type, and by age, were embedded in the tool. For example, indicator FEVE29 was restricted to fever in children aged 3 years and older, while many of the indicators were restricted to a subset of settings (e.g., TONS07 was restricted to inpatient settings as it dealt with patients having tonsillectomy or adenoidectomy).

Surveyors undertook criterion-based medical record reviews using the data collection tool. Medical records for selected visits in 2012 and 2013 were reviewed on-site at each participating facility during March–October 2016. Surveyors assessed the record for evidence that the participant presented for an indicator requiring assessment of an antibiotic overuse indicator in the years 2012 and 2013. The surveyors responded to each indicator as ‘Yes’ (care provided during the visit was consistent with the indicator), ‘No’, or ‘Not Applicable’ (NA; the indicator was not eligible for assessment in this visit). For example, a surveyor assessing an ED presentation for management of a sore throat and associated cough as required by indicator TONS04, would record ‘NA’ if the child presented with sore throat but no cough.

**Analysis**

Survey or register-derived data were used to estimate the proportion of occasions of care for each condition containing an antibiotic overuse indicator.[5-12] The number of occasions of healthcare for each condition was thereby estimated for each hospital or, for GPs and paediatricians, each health district, and sampling weights were calculated using the methods detailed in eAppendix 4 of the report of the top-line CTK results (this Appendix can be accessed by request via the corresponding author, if required).[1]

A variety of stratifications, and sometimes domain analysis,[13, 14] were necessary to ensure accuracy of the confidence interval estimates. These are detailed in eTable 2, below.

**eTable 2:**

**Domain analysis and stratifications for different estimates presented in the manuscript**

| Sub-section/Area | Domain analysis[13, 14] | Strata |
| --- | --- | --- |
| Indicator estimates | Yes | State and healthcare setting^#^ |
| Overall estimate for 3 tonsillitis indicators | Yes | Condition, state and healthcare setting^#^ |
| Overall estimate for 10 antibiotic overuse indicators | Yes | Condition, state and healthcare setting^#^ |

^#^ In SA, healthcare setting was analysed using pseudo-strata with GP and paediatrician aggregated, because all paediatricians were sampled in a single cluster, to avoid underestimation of variance.

**References:**

1. Braithwaite J, Hibbert PD, Jaffe A, White L, Cowell CT, Harris MF, et al. Quality of health care for children in Australia, 2012-2013. JAMA. 2018;319(11):1113-24.

2. Hooper TD, Hibbert PD, Mealing N, Wiles LK, Jaffe A, White L, et al. CareTrack Kids-part 2. Assessing the appropriateness of the healthcare delivered to Australian children: study protocol for a retrospective medical record review. BMJ Open. 2015;5(4):e007749.

3. Hunt TD, Ramanathan SA, Hannaford NA, Hibbert PD, Braithwaite J, Coiera E, et al. CareTrack Australia: assessing the appropriateness of adult healthcare: protocol for a retrospective medical record review. BMJ Open. 2012;2(1):e000665.

4. Runciman WB, Hunt TD, Hannaford NA, Hibbert PD, Westbrook JI, Coiera EW, et al. CareTrack: assessing the appropriateness of health care delivery in Australia. Med J Aust. 2012;197(2):100-5.

5. Britt H, Miller GC, Henderson J, Bayram C, Valenti L, Harrison C, et al. General Practice Activity in Australia 2012-13: BEACH: Bettering the Evaluation and Care of Health. Sydney, AU: Sydney University Press; 2013.

6. Harrison C. BEACH 2012-13 weighted data on frequency of management of selected conditions, for children aged 0-15, by General Practitioners. Sydney, AU: Menzies Centre for Health Policy, School of Public Health, The University of Sydney; 2017.

7. Hiscock H, Danchin MH, Efron D, Gulenc A, Hearps S, Freed GL, et al. Trends in paediatric practice in Australia: 2008 and 2013 national audits from the Australian Paediatric Research Network. J Paediatr Child Health. 2016:55-61.

8. Hiscock H. CAP 2013 data on frequency of management of selected conditions, for children aged 0-15, by paediatricians. Australian Paediatric Research Network; 2017.

9. Australian Institute of Health and Welfare. Australian hospital statistics 2012–13: Emergency Department care. Canberra, AU: AIHW; 2013.

10. Queensland Health, New South Wales Health, South Australian Department of Health. Emergency Department data on frequency of management of selected conditions, for children aged 0-15. 2017.

11. Australian Institute of Health and Welfare. Australian hospital statistics 2012–13. Canberra, AU: AIHW; 2014.

12. Australian Institute of Health and Welfare. Inpatient separations for selected conditions, as identified by ICD-10 principal diagnoses 2017. Available from: http://www.aihw.gov.au/hospitals-data/principal-diagnosis-data-cubes/.

13. Lohr S. Sampling: design and analysis. Second ed. Boston, MA: Brooks/Cole; 2009.

14. Heeringa SG, West BT, Berglund PA. Applied survey data analysis. Boca Raton, FL: CRC Press; 2010.
